# [Corrigendum] CCR7 regulates cell migration and invasion through MAPKs in metastatic squamous cell carcinoma of head and neck

**DOI:** 10.3892/ijo.2025.5761

**Published:** 2025-06-12

**Authors:** Fa-Yu Liu, Jawad Safdar, Zhen-Ning Li, Qi-Gen Fang, Xu Zhang, Zhong-Fei Xu, Chang-Fu Sun

Int J Oncol 45: 2502-2510, 2014; DOI: 10.3892/ijo.2014.2674

Following the publication of the above article, the authors drew to the Editor's attention that, for the fluorescence microscopy experiments shown in Fig. 7 on p. 2506, they had inadvertently included the same data to show the results for the 'Control' and 'CCL19 + CCR7 mAb' experiments for E-cadherin (top row, first and third images from the left). The Editorial Office subsequently conducted an independent assessment of the data in this paper and, regarding the scratch-wound assay experiments shown in Fig. 5 also on p. 2506, two pairs of the images contained overlapping sections, such that data which were intended to show the results from differently performed experiments had apparently been derived from the same original sources.

Upon examining their original data, the authors have realized that Fig. 5 was also presented incorrectly, and the revised versions of Fig. 5, now containing the correct images for the 'CCL19+PD98059 0 h', 'CCL19+SP600125 0 h' and 'CCL19+SP600125 24 h' experiments, and of Fig. 7, now showing the correct data for the E-cadherin 'Control' experiment, are shown on the next page. The authors wish to state that these errors did not affect the overall conclusions reported in the study. The authors are grateful to the Editor of *International Journal of Oncology* for allowing them this opportunity to publish a Corrigendum, and all the authors agree with its publication. Furthermore, the authors apologize to the readership for any inconvenience caused.

## Figures and Tables

**Figure 5:**
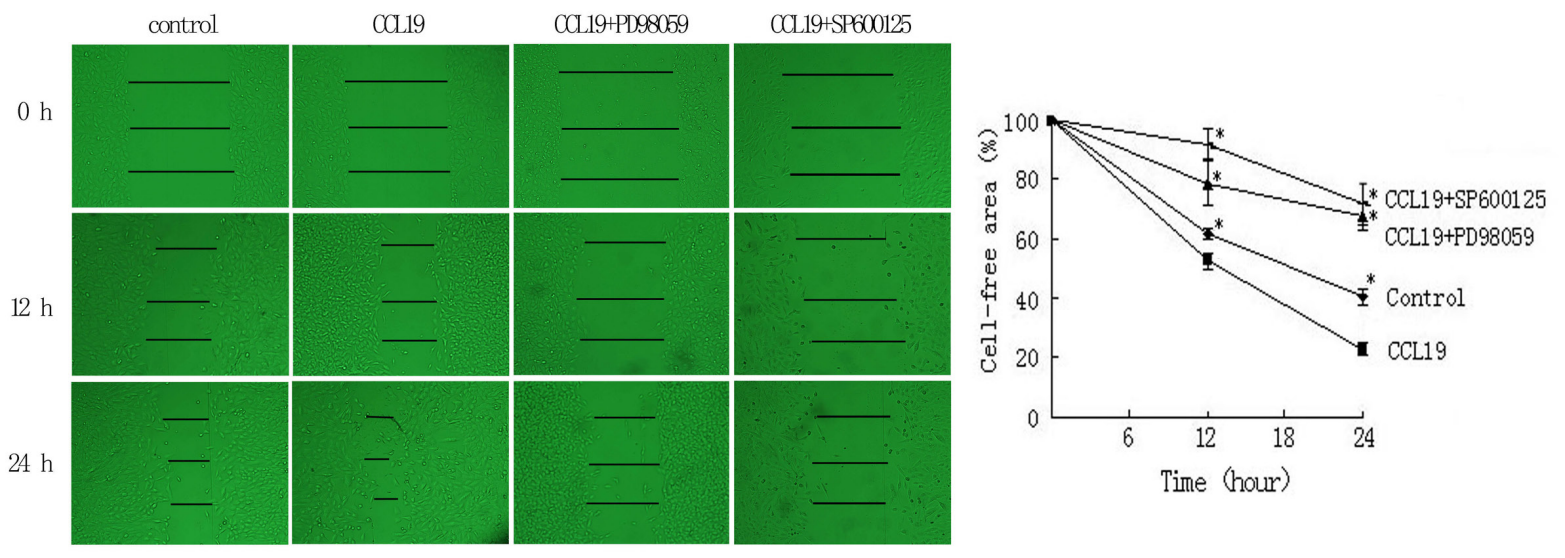
ERK1/2 and JNK inhibitors in CCL19-induced wound-healing. PCI-37B cells were pre-treated with PD98059 (20 μM) and SP600125 (50 μM) for 4 h, and then CCL19 (500 ng/ml), the cell free area was evaluated at 0–24 h. The results are representative of three independent experiments. ^*^P<0.05 compared to CCL19 group.

**Figure 7:**
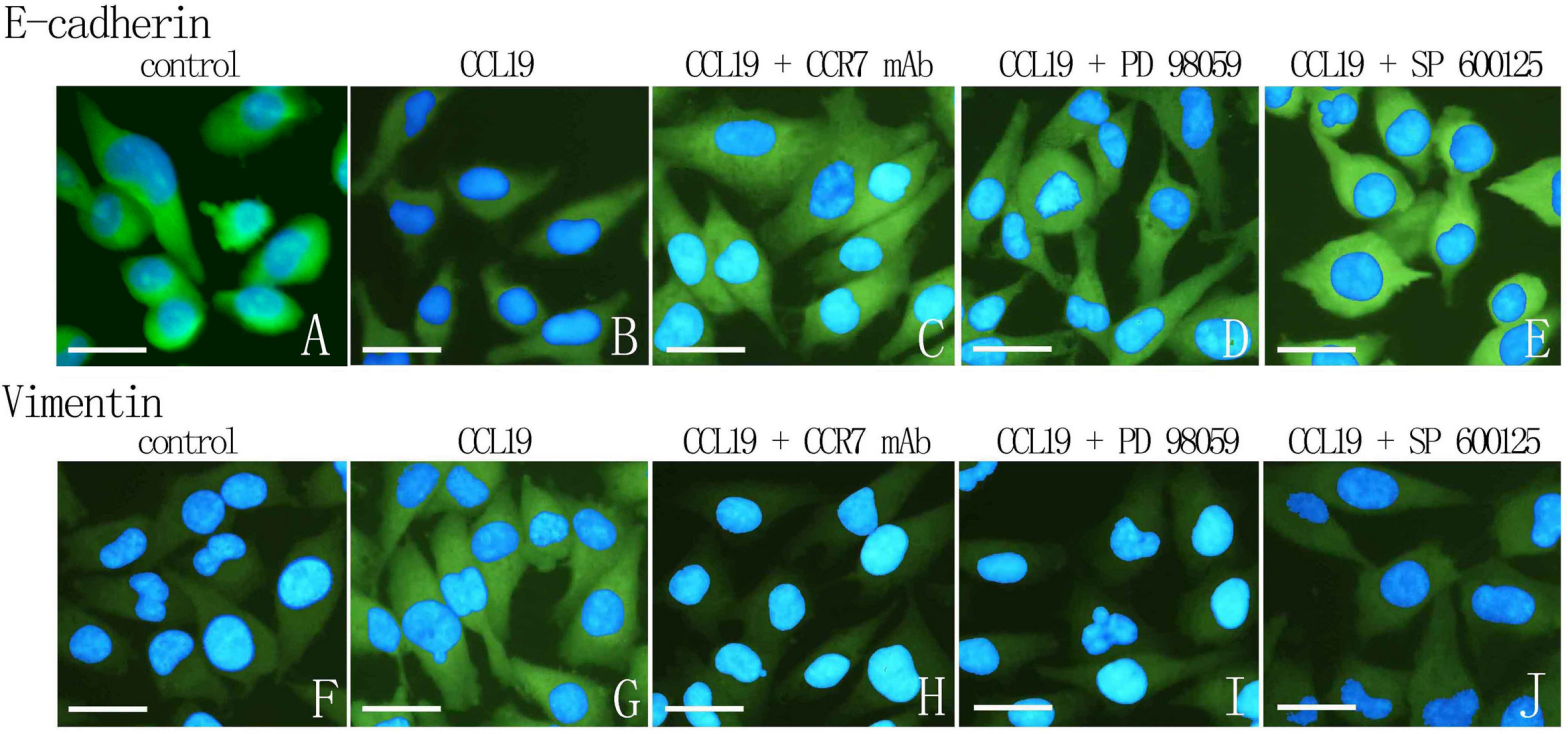
CC chemokine receptor 7 (CCR7), ERK1/2 and JNK in CCL19-induced E-cadherin and Vimentin distribution. PCI-37B cells were pre-treated with CCR7 mAb (10 μg/ml), PD98059 (20 μM) and SP600125 (50 μM) for 4 h, and then CCL19 (500 ng/ml, 30 min). E-cadherin and Vimentin distribution was assessed by immunostaining and fluorescence microscopy. The results are representative of three independent experiments.

